# The Uganda – Democratic Republic of the Congo cross-border collaboration for Bundibugyo Ebola response: A new model for African health security, sovereignty and solidarity

**DOI:** 10.4102/jphia.v17i1.2180

**Published:** 2026-07-31

**Authors:** Henry K. Bosa, Pierre Z. Akilimali, Ingrid Ampeire, Atek Kagirita, Charles Olaro, Dieudonné Mwamba, Diana Atwine, Kasonde Mwinga, Christian Ngandu, Wessam Mankoula, Catherine A.H. Smallwood, Nebiyu Dereje, Marie-Roseline Belizaire, Yap Boum, Mohamed Y. Janabi, Jean Kaseya, Chris Baryomunsi, Samuel R. Kamba

**Affiliations:** 1 Ministry of Health of Uganda, Kampala, Uganda; 2 Ministry of Public Health, Hygiene and Social Welfare of Democratic Republic of the Congo, Kinshasa, Democratic Republic of the Congo; 3 Uganda Country Office, World Health Organization, Kampala, Uganda; 4 Africa Centres for Disease Control and Prevention, Addis Ababa, Ethiopia; 5 School of Public Health, Wachemo University, Hosainna, Ethiopia; 6 World Health Organization Africa Region, Brazzaville, Democratic Republic of the Congo

The ongoing Bundibugyo Ebola virus disease outbreak in eastern Democratic Republic of the Congo (DRC) is a real-world demonstration of a new African approach to epidemic preparedness and response, founded on three complementary principles: health security, health sovereignty and solidarity. While these concepts have increasingly guided continental health policy through the Africa Health Security and Sovereignty (AHSS) agenda,^1^ the current outbreak represents one of the first opportunities to translate these principles into operational reality at the regional level. The current outbreak was first detected in Mongwalu, Ituri province, a region where the border between Uganda and the DRC has always been more administrative than social. Communities on both sides share ethnic ties, languages, markets, schools, families and health services. Every day, thousands of people cross the border to trade, seek healthcare, attend school or visit relatives. Consequently, pathogens move through the same networks that sustain daily life, heightening the need for cross-border collaboration and response efforts.^2^

Historically, outbreaks have often prompted countries to close borders or impose strict travel restrictions to contain transmission. While such measures may temporarily reduce movement, they can also disrupt livelihoods, delay access to healthcare, separate families, disrupt humanitarian assistance and encourage informal crossings that are considerably harder to monitor.^3^ Cross-border collaboration has proven essential in controlling Ebola outbreaks in the past. During the 2014–2016 West African epidemic, most transmission across Guinea, Liberia and Sierra Leone occurred over land where airport screening had little effect. Community-level cross-border collaboration, including joint surveillance and engagement of traditional leaders, was later identified as key to controlling the outbreak.

Cognisant of this, Uganda and the DRC have chosen cooperation rather than isolation. Instead of treating the international border as a barrier, the two countries are transforming it into a platform for coordinated public health action. Moreover, as demonstrated in the mpox response, a unified Incident Management Support Team (IMST) has been established, jointly led by the Africa Centres for Disease Control and Prevention (Africa CDC) and the World Health Organization (WHO), to coordinate the response.^4,5^ The IMST brings together the affected countries, at-risk countries and partners, ensuring efficiency and avoiding duplication of response activities.

The cross-border collaboration also led to the establishment of joint mobile Ebola Treatment Centres (ETCs) in Aru and Kasenyi on the Congolese side of the border, supported by joint mobile laboratories capable of providing rapid diagnosis close to affected communities. These ETCs and mobile laboratories are staffed by healthcare workers from DRC and Uganda working together, sharing expertise, equipment and operational experience while ensuring that patients receive timely diagnosis and treatment where they are most needed. This approach substantially shortens laboratory turnaround times, accelerates case confirmation, facilitates rapid isolation, strengthens contact tracing and improves clinical management. At the same time, it reinforces confidence among healthcare workers and affected communities by bringing life-saving services closer to populations living in remote border areas.

At the heart of this collaboration was a group of public health experts willing to work together and share response strategies. Early meetings held in Aru laid the groundwork for trust and mutual respect between the Congolese and Ugandan teams. This included respect not only for national sovereignty, but also for the Ebola expertise and experience present on both sides of the border. Building on this foundation, meetings between the teams, held informally under mango trees along the border, enabled the exchange of real-time public health intelligence. This exchange quickly resolved the case of a Ugandan patient who had gone missing from follow-up and prevented onward transmission from a Congolese patient who had sought early care on the Ugandan side of the border. Within weeks, this informal partnership had matured from a concept to functioning cross-border collaborative surveillance.^6^

These operational innovations have been formalised through a bilateral Memorandum of Understanding (MoU) that establishes an innovative governance framework for cross-border epidemic preparedness and response. More than a formal agreement, the Memorandum operationalises three complementary principles that together may define a new African model for managing cross-border public health threats.^7^ The MoU was launched by delegates from both countries, together with partners, in Aru on 23 June 2026.

The first principle is health security. Rather than protecting each country independently, the DRC and Uganda are strengthening regional health security through coordinated surveillance, harmonised case definitions, interoperable laboratory networks, joint treatment capacity, synchronised contact tracing and real-time information sharing. This approach recognises that the health security of one country depends directly on the preparedness and response capacity of its neighbour. Protecting one side of the border without protecting the other is neither sustainable nor effective.^8^

The second principle is health sovereignty. Cross-border collaboration does not diminish national authority. Instead, epidemiological information, laboratory samples, biological specimens and public health decision-making remain under the ownership and authority of the DRC while benefiting from shared technical expertise and operational support from Uganda and continental institutions. This distinction is particularly important because previous international emergency responses have sometimes generated concerns regarding external ownership of national response efforts. The current model demonstrates that regional cooperation can reinforce, rather than replace, national leadership.^9,10^

The third principle is solidarity, inspired by the African philosophy of Ubuntu: ‘*I am because we are*’.^11^ This solidarity is expressed not only through political commitments but also through daily operational practice. Laboratory scientists, epidemiologists, clinicians, nurses, logisticians, surveillance officers, infection prevention specialists and emergency managers from both countries are working side by side within the same treatment centres, laboratories and coordination mechanisms. Their collaboration simultaneously strengthens the Ebola response, transfers knowledge, builds local expertise and reinforces health-system capacity in one of the continent’s most vulnerable border regions.

Digital innovation is further strengthening this partnership. Cross-border points of entry between Aru and Arua are progressively being equipped with interoperable digital screening systems that allow real-time exchange of traveller information, contact lists and surveillance data. These systems facilitate rapid identification and follow-up of high-risk contacts, reduce duplication of efforts and improve situational awareness for decision-makers on both sides of the border. Digitalisation is therefore becoming an essential enabler of coordinated regional surveillance. The governance architecture supporting these innovations extends beyond bilateral collaboration. Through the joint Africa CDC–WHO Continental IMST, technical leadership, operational planning and partner coordination are increasingly aligned under a common incident management framework. This model seeks to overcome one of the major challenges observed during previous outbreaks, namely the disconnect between strategic coordination at the continental level and operational implementation in affected countries. By integrating leadership, planning, logistics, surveillance, laboratory services, case management and cross-border preparedness within a unified structure, the IMST is helping translate continental solidarity into operational effectiveness.

The official launch of this cross-border collaboration, therefore, represents much more than the inauguration of treatment centres and laboratories. It demonstrates that African leadership can transform a health crisis into an opportunity for regional integration. Rather than allowing borders to become barriers, the response is bringing neighbouring countries closer together through trust, shared responsibility, transparency and mutual accountability. This coordination and governance approach can also be scaled up to support preparedness and response for cholera, mpox, Marburg virus disease, Rift Valley fever and other epidemic-prone diseases that repeatedly affect African border regions.

Sustaining and scaling up this cross-border collaboration depends on implementing five key priorities. Firstly, it is critical to expand and institutionalise cross-border coordination mechanisms. The Uganda–DRC experience has demonstrated the value of joint planning, information sharing and coordinated response actions. Sustaining these gains will require extending the model to additional high-risk border areas such as Kasenyi and Chomia, strengthening cross-border governance structures, and maintaining political commitment to regional solidarity and joint decision-making. Effective preparedness must remain grounded in shared responsibility while respecting national sovereignty.

Secondly, strengthening the collaborative surveillance approach is essential if public health intelligence is to be shared in real-time across national borders. The ongoing outbreak has highlighted the importance of interoperable surveillance systems capable of tracking cases, contacts, population movement and emerging risks across borders. Future efforts should focus on harmonising surveillance protocols, strengthening community-based surveillance, integrating mobility and epidemiological data, and building routine cross-border exchange of real-time public health intelligence to support early detection and rapid response to all priority diseases.

Thirdly, it is important to enhance joint laboratory and diagnostic capacity. Rapid diagnosis remains central to outbreak control. Strengthening laboratory networks, specimen referral systems, diagnostic interoperability and surge testing capacity across border regions will improve the speed and effectiveness of response efforts. Investments in shared laboratory preparedness can also support broader regional health security objectives beyond Ebola.

Fourthly, institutionalising preparedness through joint simulation exercises and workforce development is critical. Regular joint simulation exercises, after-action reviews and cross-border training programmes are critical to maintaining operational readiness. Institutionalising these activities will help countries test preparedness plans, identify response gaps, strengthen sectoral coordination and build a workforce capable of responding effectively to future public health emergencies.

Fifthly, generating evidence and scaling lessons across Africa is indispensable. Rigorous evidence generation and documentation of the Uganda–DRC collaboration is essential to demonstrate the added value of integrated cross-border preparedness. The lessons learned should be systematically captured and translated into policy guidance that can inform replication in other high-risk regions. By serving as a proof of concept for integrated public health action, this model has the potential to become a cornerstone of Africa’s future epidemic preparedness and emergency management architecture.
